# A Meta-Analysis of Choroidal Thickness Changes in Unilateral Amblyopia

**DOI:** 10.1155/2017/2915261

**Published:** 2017-06-19

**Authors:** Yanli Liu, Yi Dong, Kanxing Zhao

**Affiliations:** ^1^Clinical College of Ophthalmology, Tianjin Medical University, 4 Gansu Road, Tianjin 300020, China; ^2^Tianjin Baodi Hospital, Baodi Clinical College of Tianjin Medical University, 8 Guangchuan Road, Tianjin 301800, China; ^3^Tianjin Eye Hospital, 4 Gansu Road, Tianjin 300020, China; ^4^Tianjin Key Laboratory of Ophthalmology and Visual Science, 4 Gansu Road, Tianjin 300020, China; ^5^Tianjin Eye Institute, 4 Gansu Road, Tianjin 300020, China

## Abstract

**Purpose:**

To date, the topic of amblyopic changes remains controversial. Therefore, a systematic review and meta-analysis were carried out to evaluate choroidal changes in unilateral amblyopia.

**Methods:**

Major literature databases were searched for amblyopia-relevant studies. Using enhanced depth imaging optical coherence tomography (EDI-OCT), the primary outcome parameters examined were subfoveal choroidal thickness (SFCT) and different choroidal thickness (CT) positions. Efficacy estimates were evaluated by weighted mean difference (WMD) and 95% confidence interval (CI) for choroidal-associated changes. We performed subgroup analysis and metaregression analysis to examine potential sources of heterogeneity.

**Results:**

Eleven cross-sectional studies that included a total of 768 participants were identified. The amblyopic eye SFCT was thicker than that of the fellow and control (normal) eyes (WMD_amblyopia versus fellow_ = 49.24, 95% CI of 30.22 to 68.27, *p* < 0.001; WMD_amblyopia versus control_ = 54.51, 95% CI of 32.17 to 76.85, *p* < 0.001). There were no differences between the fellow and control eyes (WMD = 13.81, 95% CI of 1.16 to 28.77, *p* = 0.071). Subgroup and metaregression analyses indicated that the OCT type was the main source of heterogeneity.

**Conclusions:**

The CT in the amblyopic eyes was thicker than that in the fellow and control eyes.

## 1. Introduction

With a prevalence of 1%–3.5%, amblyopia is the most common cause of unilateral vision impairment in children and young adults. The main causes include anisometropia, strabismus, or a combination of both factors. The amblyopic process may involve various levels of the visual pathway such as the extensive visual cortex, lateral geniculate nucleus, and retina. The development of enhanced depth imaging optical coherence tomography (EDI-OCT) has allowed in vivo cross-sectional choroidal imaging and precise quantitative analysis of choroidal thickness (CT). In different experimental animal models, the choroid has been shown to be associated with the development of the refractive state and axial elongation. The choroid is another region in the amblyopia that is currently being investigated. In recent years, numerous articles using EDI-OCT technology have been published in which choroidal thickness has been evaluated in unilateral amblyopia. There is no consensus about whether the amblyopic choroid is structurally abnormal. Given the inconsistency among the current publications and insufficient statistical power of primary trials, we used a meta-analysis to examine the existing evidence obtained by EDI-OCT for detecting choroidal changes in unilateral amblyopia.

## 2. Methods

### 2.1. Literature Search

This meta-analysis was performed according to a predefined protocol that is described below. Two investigators independently searched PubMed, Embase, Web of Science, ClinicalTrials.gov, and the Cochrane Central Register of Controlled Trials databases for publications (accessed on September 20, 2016). The search algorithm was designed in a specific format. We used combined terms for either MeSH or title/abstract relating to “amblyopia or amblyopi^*^” and “OCT or optical coherence tomography” and choroidal thickness. We also searched the internet using the Google Scholar search engine to obtain information. Using PubMed as an example, the specific search strategy is depicted in an Additional File 2 available online at https://doi.org/10.1155/2017/2915261. Additional studies were identified via a manual reference search of the original studies and review articles. We retrieved electronic searches to identify studies not yet included in the computerized databases. No language restriction in the search process was used, and all studies performed on human subjects were included in our search.

### 2.2. Inclusion and Exclusion Criteria

Published clinical trials were included if they fulfilled all of the following criteria: (1) they evaluated CT (subfoveal or around the fovea area) using spectral domain OCT with the EDI program; (2) patients with unilateral amblyopia were included, and the study compared the amblyopic eye with the fellow or with the visually normal control eyes. Amblyopia was defined as the best-corrected visual acuity (BCVA) of ≤20/30 in one eye or at least two lines worse than that in the fellow normal eye. Amblyopia was classified as anisometropic and/or strabismic amblyopia with cycloplegic refraction >2 diopters; (3) cases were of cross-sectional, case-control, or case-series design; and (4) at least one of the relevant outcome variables discussed subsequently was included. We initially scrutinized the titles and abstracts of all references and then rescreened full-text papers. Articles were excluded if they did not mention OCT or describe patients' refractive status.

### 2.3. Data Extraction and Clinical Outcome

Data extraction was performed independently by two authors according to a customized protocol, and discrepancies were mediated by a third reviewer. A standard data collection form was used when data extraction was performed. Several pieces of information were extracted from the included trials: (1) author name; (2) year of publication; (3) location of trials; (4) number of subjects; (5) mean age; (6) type of amblyopia; (7) type of OCT; and (8) axial length (AL). The primary outcome parameters investigated in this study were mean CT, mean AL, and age of each group. It was determined whether the AL was or was not matched according to AL matching status in the studies. CT as measured by EDI-OCT was recorded for each group at different macular points. CT was defined as the vertical distance from the outer portion of the hyperreflective line (corresponding to Bruch's membrane beneath the retinal pigment epithelium) to the outermost hyperreflective line of the inner scleral border. The mean of these values was used to calculate CT.

### 2.4. Quality Assessment

The methodological quality of the studies included was assessed using an 11-item checklist, which was based on the scale of the Agency for Healthcare Research and Quality (AHRQ). An item would be scored “0” if the answer was “NO” or “UNCLEAR”; if the answer was “YES”, then the item would be scored “1”. The article quality was assessed as follows: (1) low quality = 0–3; (2) moderate quality = 4–7; or (3) high quality = 8–11.

### 2.5. Statistical Analysis

Analyses were performed using STATA version 12.0 (StataCorp LP, College Station, TX, USA), and values of *p* < 0.05 were considered statistically significant. For continuous outcomes, we quantified with weighted mean difference (WMD) and their 95% confidence intervals. The outcomes were measured as mean ± SD. Heterogeneity between the studies included in the meta-analysis was assessed and quantified using the chi square-based Q statistic test and the *I*^2^ metric. Findings were taken to be statistically significant if *p*_Q_ ≤ 0.10 or *I*^2^ > 50%. If there was statistically significant heterogeneity among studies, a random-effect model was used. Alternatively, data were pooled using a fixed-effect model. We used both a univariate and multivariate metaregression model to conduct the metaregression. To tackle the issue of multiple testing, 10,000 permutations of Monte Carlo simulation needed to be carried out to adjust the result of the multivariate metaregression model. In addition, publication bias was evaluated by Begg's rank correlation and Egger's linear regression tests. Two-tailed *p* values <0.05 were considered to be statistically significant. Result reliability was assessed by sensitivity analysis performed by sequentially omitting individual studies.

## 3. Results

### 3.1. Search and Selection of Studies

**Figure 1:**
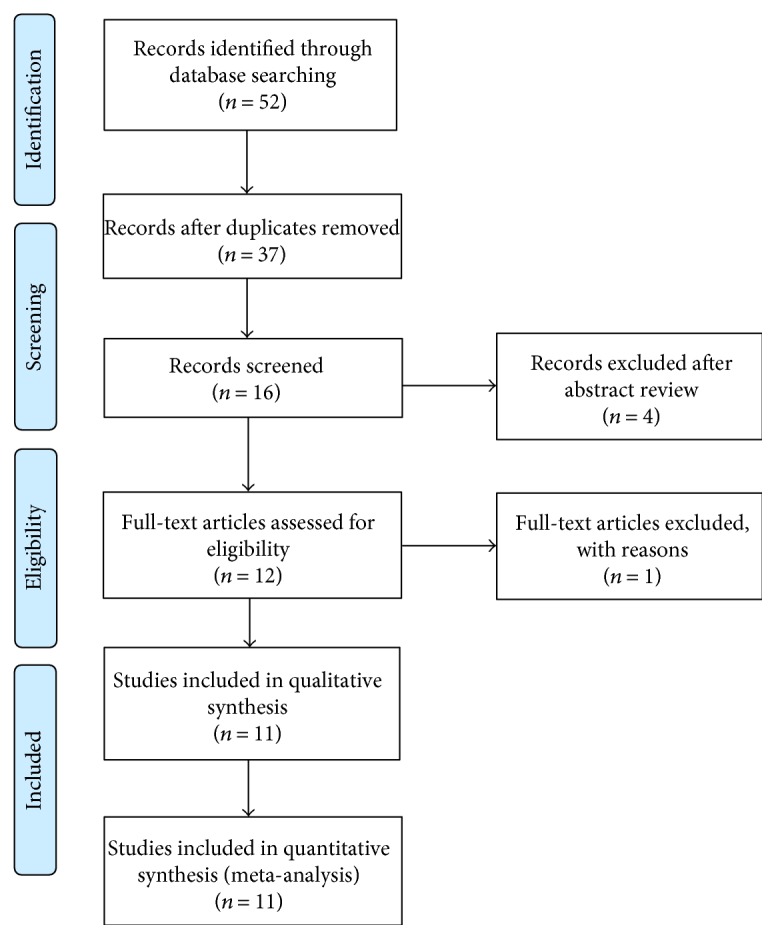
PRISMA flow diagram of studies included in the meta-analysis.

We initially identified 52 potential studies. After duplicates had been removed, the number was 37. The majority were excluded after the first screening based on abstracts or titles, primarily because they were reviews, case reports, letters, or not relevant to our analysis. Four studies were excluded because their information was incomplete or irrelevant. In a study by Wan et al. [1], the spherical equivalent was more than −6 D and AL was beyond 26 mm, which may have included pathological myopia; thus, we excluded it. A total of 11 studies were retained for the meta-analysis. A flow chart showing the study selection is presented in Figure 1.

A total of 52 articles were identified from the databases, and 15 duplicates were excluded. Twenty-one articles were excluded based on a review of the titles and abstracts, and 12 full-text articles were assessed for eligibility; one article was excluded due to various reasons. Finally, a total of 11 articles were included in this meta-analysis.

### 3.2. Characteristics and Quality of the Trials

There were 449 patients included in this study. Of the 11 enrolled publications, nine studies compared the amblyopic eyes with the normal control eyes and 10 studies compared the amblyopic eyes with the fellow eyes. These studies were performed in three Asian countries. These trials were reported between 2014 and 2016. The range of average ages was from 4 to 65 years. Eleven studies used EDI-OCT technology to diagnose amblyopia. Details of each study, such as age, type of amblyopia, OCT type, and methodological quality assessment, are presented in Table 1.

### 3.3. Meta-Analysis

#### 3.3.1. Changes in Subfoveal Choroidal Thickness

**Figure 2:**
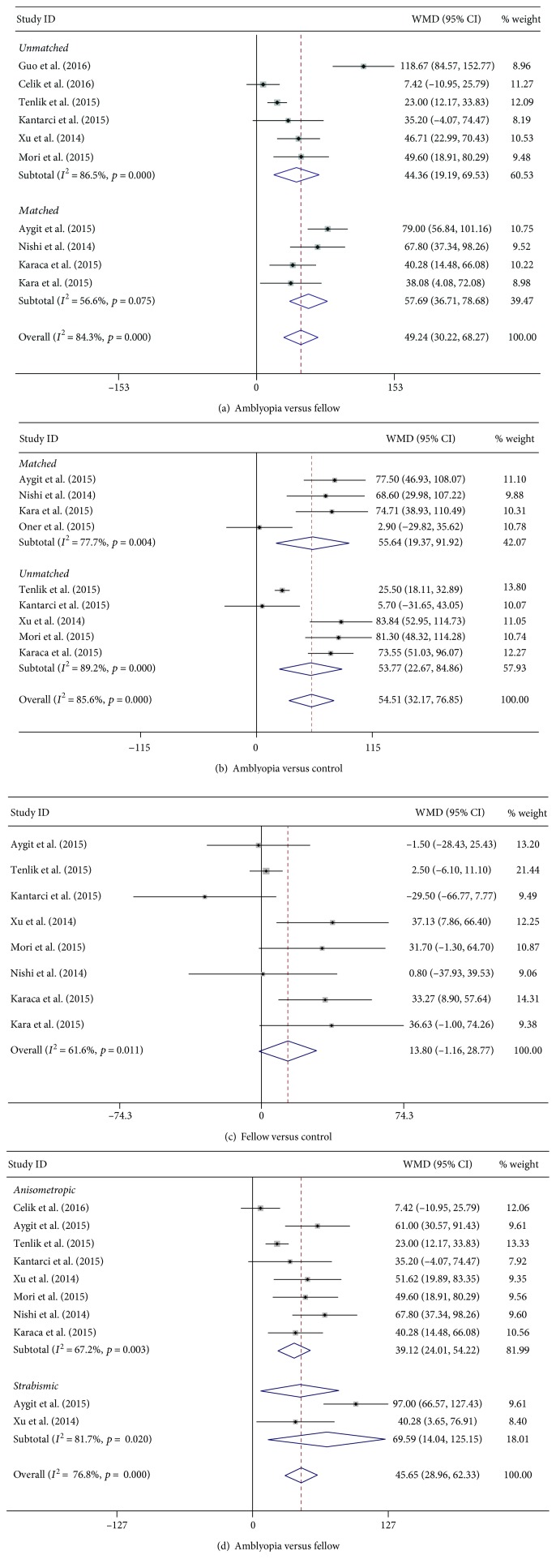
Pooled estimates for all studies comparing SFCT in the amblyopic eye with the fellow eye and the normal control eyes. (a) The amblyopic eye versus the fellow eye. (b) The amblyopic eye versus the control eye. (c) Fellow versus control. (d) Amblyopic type subgroup analysis when the amblyopic eye is compared to the fellow eye. The SFCT of the amblyopic eye was thicker than that of both the fellow and the control eyes, both in the overall and in the subgroups (AL matched/not). The results for the amblyopic type subgroup analysis were the same as for the axial length subgroup analysis. Note: weights are from random effects analysis.

As shown in Table 2 and Figure 2(), the subfoveal choroidal thickness (SFCT) of the amblyopic eye was greater than that of the fellow and control eyes (WMD_amblyopia versus fellow_ = 49.24, 95% CI of 30.22 to 68.27, *p* < 0.001; WMD_amblyopia versus control_ = 54.51, 95% CI of 32.17 to 76.85, *p* < 0.001). There were no differences between the fellow and the control eyes (WMD = 13.81, 95% CI of −1.16 to 28.77, *p* = 0.071). According to whether the axial length was matched or not, we divided all the studies into two subgroups and conducted subgroup analysis. The SFCT of the amblyopic eye was thicker than that of the fellow and control eyes, whether in the matched group or in the unmatched group (Table 2). In addition, the results for the amblyopia type subgroup analysis were the same as for the axial length subgroup analysis (Figure 2(d)).

The SFCT of the amblyopic eye was thicker than that of the fellow eye and the control eye, both in the overall and in the subgroups (whether AL was matched or not). No evidence of publication bias was found using Begg's or Egger's tests. There was obvious heterogeneity, both in the overall and in the subgroups.

#### 3.3.2. Changes in Choroidal Thickness at Other Positions

In these studies, CT was measured at ten different positions (shown in Table 3). Among these, the temporal 1.0 mm and the nasal 1.0 mm cavities are the points of interest. The CT in the temporal 1.0 mm of the amblyopic eye was thicker than that of the fellow and control eyes (WMD_amblyopia versus fellow_ = 20.83, 95% CI of 11.01 to 30.64, *p* < 0.001; WMD_amblyopia versus control_ = 29.01, 95% CI of 15.01 to 49.00, *p* < 0.001). Moreover, CT in the nasal 1.0 mm cavity of the amblyopic eye was thicker than that of the fellow and control eyes (WMD_amblyopia versus fellow_ = 36.95, 95% CI of 20.52 to 53.38, *p* < 0.001; WMD_amblyopia versus control_ = 55.05, 95% CI of 28.60 to 81.50, *p* < 0.001). There were no differences between the fellow and control eyes. However, the CT values in the temporal 0.5 mm, nasal 0.5 mm, superior 1.0 mm, inferior 1.0 mm, temporal 2.0 mm, nasal 2.0 mm, temporal 3.0 mm, and nasal 3.0 mm were measured in only two studies.

The CT in other positions of the amblyopic eye was thicker than that of the fellow and control eyes.

### 3.4. Metaregression Analysis

To examine the possible sources of heterogeneity, studies were stratified by eye axial length that was either matched or not matched, but the heterogeneity did not decrease. We therefore performed a metaregression analysis to assess the influence of the study characteristics on the meta-analysis. The eye axial length, matched/not matched, and the OCT type were treated as suspect factors. Metaregression analysis showed that the OCT type was the main source of heterogeneity (*p* < 0.001 for univariate metaregression and *p* = 0.003 for multivariate metaregression, after 10,000 permutations of Monte Carlo simulation).

### 3.5. Sensitivity Analysis and Publication Bias

**Figure 3:**
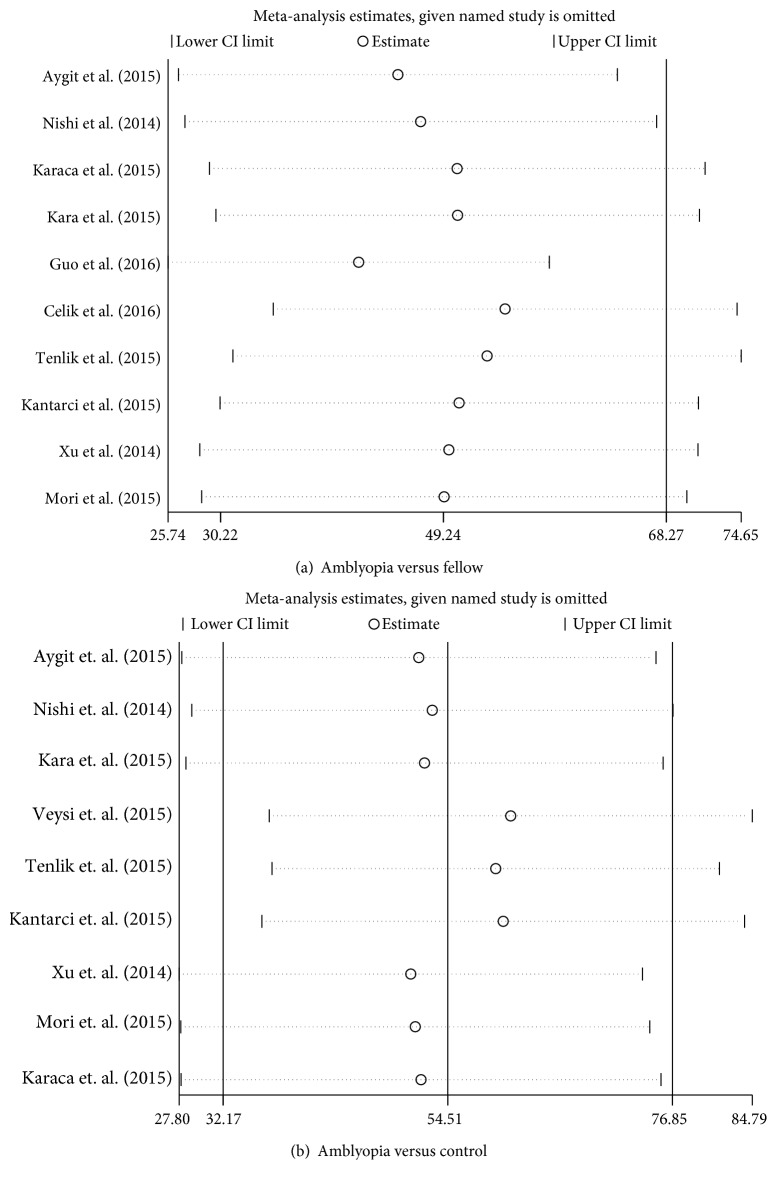
Sensitivity analysis of meta-analysis. (a) The amblyopic eye versus the fellow eye. (b) The amblyopic eye versus the control eye. When one study is deleted at a time, the corresponding estimates do not change significantly.

To evaluate the influence of an individual data set on the pooled results, one study was deleted at a time. The corresponding estimates did not change greatly when any single study was deleted, thus indicating the high stability and reliability of the meta-analysis results (Figure 3). Publication bias was tested using Begg's and Egger's tests, and no obvious evidence of publication bias was found (Tables 2 and 3).

## 4. Discussion

This meta-analysis of 11 clinical trials involving 449 patients indicated that the amblyopic process may involve the choroid. Our results showed that the CT was thicker in the amblyopic eyes than in both the fellow and control eyes. The results did not indicate that SFCT differed between anisometropic and strabismic amblyopia. The majority of previous studies had been limited by relatively small sample sizes, and only some of them had healthy control subjects. Because of insufficient statistical power in previous studies, our analysis contained a large number of patients, which increased the power to provide more convincing assessments.

Based on our meta-analysis, CT was significantly thicker in the amblyopic eye than in the fellow and control eyes from the fovea to the temporal and nasal cavities at different interval (range: 0.5 mm~3 mm) points. Our analysis did not find a significant difference between the fellow and control eyes. Anatomically, the retinal fovea is nourished by the choroidal blood vessel. Moreover, the foveola was thicker in the amblyopic eyes than that in the visually normal control eyes. A thicker retina may require additional blood for nourishment. Therefore, the CT may have to increase to supply additional blood to the retina. Increased choroidal blood flow can cause choroidal thickening.

Kara et al. [12] thought that amblyopia affected the process of postnatal maturation of the choroids and that amblyopia was associated with changes in choroidal function in humans. With split-spectrum amplitude-decorrelation angiography OCT, Guo et al. [2] discovered that 86.4% of the amblyopic eyes displayed a blurry choroidocapillary network and that 68.2% of the amblyopic eyes had a dark atrophic patch. It was possible that the choroidocapillary atrophy patch caused a wide range of compensatory dilatation of its surrounding capillaries, and because of this, blurry images occurred. Dilatation of the choroidal vessels may lead to increased CT.

The choroid plays a role in emmetropization and refractive error development in animal species and humans and has been shown to be involved in the visual feedback pathway. The choroid becomes thicker with myopic defocus (image in front of the retina) and thinner with hyperopic defocus (image behind the retina), even within hours, which in turn adjusts the position of the retina to maintain clear vision. Troilo et al. [13] suggested that the thickened choroid might retard the growth of the eye during development, as a result of providing a barrier to diffusion of growth factors or a mechanical buffer to limit the eye's elongation. Nishi et al. [9] hypothesized that the choroidal changes in CT in response to defocus did not occur in the hyperopic amblyopic eyes; thus, the subfoveal CT was thicker and ocular growth was limited. Myopic defocus can cause choroid thickening. Several clinical trials have shown that a myopic defocus ring in the peripheral retina can slow axial growth; this may be caused by orthokeratology or spherical and multifocal soft contact lenses.

However, no studies have been done to show whether a cause-and-effect relationship exists between amblyopia and thickened CT. It fails to explain the findings in which thicker choroids in the strabismic amblyopic eyes exist without any differences in refractive error between the two eyes. Additional clinical studies are required to elucidate the differences.

Recent studies have shown variability in choroidal thickness in the healthy eyes according to axial length, age, and diurnal rhythms, which results in heterogeneity. In the present meta-analysis, the mean age of the included subjects in different studies ranged from 4.5 to 39.5 years, which might have affected the pooled results. In children, AL changes with eye growth. At the same time, choroidal thickness increases significantly from early childhood to adolescence in normal pediatric subjects. In contrast, previous studies reported that SFCT negatively correlated with age and SFCT decreased 15.6 *μ*m for each decade of life. In the amblyopic eyes, some studies showed CT negatively correlated with AL, while others were opposite. Therefore, we used a subgroup analysis according to whether or not AL was matched, with the fellow and/or age-matched control eyes. Both subgroups showed a similar result, in which the SFCT for the amblyopic eyes was significantly thicker than that for the fellow and control eyes. In two studies with unmatched AL, the results did not change after adjusting for the axial length using a generalized estimating equation. All the OCT measurements were taken at the same time within each enrolled publication, but the time was different between each study, which would have introduced heterogeneity. Instrument error and differences in examination instruments could also have affected the results. Our metaregression analysis result is similar to that of theirs and the OCT type was the main source of heterogeneity. However, previous studies proved that the CT measurements obtained with different SD-OCTs were significantly correlated and could be used interchangeably.

There were certain limitations to our study. Firstly, this meta-analysis did not include studies using swept-source OCT, which is the latest milestone in choroidal imaging. However, we could not search any data about CT of the amblyopic eyes involving this technique. Secondly, we did not compare choroidal structural differences among subjects with persistent and resolved amblyopia or those with treated and untreated amblyopia. In addition, the difference in the unilateral deprivational amblyopic eyes was not evaluated, owing to lack of data. Thirdly, the study did not demonstrate a clear causal relationship between a thickened choroid and the amblyopia, because all the included trials were observational studies. Fourthly, in the meta-analysis, substantial heterogeneity was observed among the studies. However, we performed subgroup and sensitivity analyses in which the stability and reliability were high. Finally, given that factors other than AL and age most likely affect choroidal thickness, our conclusions should be evaluated with caution.

## 5. Conclusion

In Asian populations, CT in the amblyopic eyes was thicker than that in the fellow and normal control eyes. Further studies are needed to confirm the clear causal relationship between thickened choroid and amblyopia and the mechanism involved, by the same type of OCT.

## Supplementary Material

Additional file 1. PRISMA 2009 checklist. Additional file 2. search strategy (PubMed).

## Figures and Tables

**Table 1:** Characteristics of the 11 studies included in the analysis.

Study	Location	Amblyopia	Sample size		Age (years)		Type of OCT	AHRQ score
			Case	Control	Case	Control		
Guo et al. [2]	China	A	13	—	9.6 ± 2.45	—	Spectralis OCT	8
		S	9					
Celik et al. [3]	Turkey	A	43	—	24.8 ± 7.4	—	Cirrus HD-OCT	8
Aygit et al. [4]	Turkey	A	40	40	7.9 ± 2.6	8.4 ± 2.6	Spectralis OCT	9
		S	40		9.0 ± 3.7			
Tenlik et al. [5]	Turkey	A	53	53	17.8 ± 11.0	17.7 ± 11.0	Cirrus HD-OCT	9
Kantarci et al. [6]	Turkey	A	54	52	39.5 ± 10.4	38.5 ± 10.2	RS-3000 OCT	7
Xu et al. [7]	China	A	21	22	7.86 ± 1.85	7.82 ± 1.10	Spectralis OCT	8
		S	16		9.13 ± 2.00			
Mori et al. [8]	Japan	A	24	12	4.5 ± 1.4	Matched	Spectralis OCT	9
Nishi et al. [9]	Japan	A	25	20	6.7 ± 1.9	6.6 ± 2.2	Spectralis OCT	9
Öner et al. [10]	Turkey	A	33	42	10.7 ± 3.3	11.2 ± 3.3	Cirrus HD-OCT	8
Karaca et al. [11]	Turkey	A	40	40	8.82 ± 3.6	9.45 ± 2.25	Spectralis OCT	8
Kara et al. [12]	Turkey	A	17	38	10.00 ± 2.87	11.84 ± 3.17	Spectralis OCT	8

S: strabismic amblyopia; A: anisometropic amblyopia; AL: axial length; AHRQ: Agency for Healthcare Research and Quality.

**Table 2:** Pooled results comparing SFCT in the amblyopic eye with the fellow eye and the normal control eye.

Groups	AL/*n*	WMD (95% CI)	*p* value	Test for heterogeneity	Egger's test (*p*)	Begg's test (*p*)
A versus F	All/10	49.24 (30.22–68.27)	*p* < 0.001	*I* ^2^ = 84.3%, *p* < 0.001	0.291	0.622
	Matched/4	57.69 (36.71–78.68)	*p* < 0.001	*I* ^2^ = 56.6%, *p* = 0.075	0.410	0.497
	Unmatched/6	44.36 (19.19–69.53)	*p* = 0.001	*I* ^2^ = 86.5%, *p* < 0.001	0.175	0.348

A versus C	All/9	54.51 (32.17–76.85)	*p* < 0.001	*I* ^2^ = 85.6%, *p* < 0.001	0.093	0.427
	Matched/4	55.65 (19.37–91.92)	*p* = 0.003	*I* ^2^ = 77.7%, *p* = 0.004	0.851	0.497
	Unmatched/5	53.77 (22.67–84.86)	*p* = 0.001	*I* ^2^ = 89.2%, *p* < 0.001	0.195	0.624

F versus C	All/8	13.80 (−1.16, 28.77)	*p* = 0.071	*I* ^2^ = 61.6%, *p* = 0.011	0.340	1.000

SFCT: subfoveal choroidal thickness; AL: axial length; WMD: weighted mean differences; CI: confidence interval; A: amblyopia; C: control; F: fellow.

**Table 3:** Pooled estimates of all studies comparing other positions in the amblyopic eye with the fellow and normal eyes.

Group	Position/*n*	WMD (random) (95% CI)	*p* value	Test for heterogeneity	Egger's test (*p*)	Begg's test (*p*)
A versus F	T 0.5 mm/2	40.14 (15.00–65.27)	*p* = 0.002	*I* ^2^ = 0.0%, *p* = 0.577	—	0.317
	N 0.5 mm/2	46.38 (20.35–72.42)	*p* < 0.001	*I* ^2^ = 0.0%, *p* = 0.566	—	0.317
	T 1.0 mm/6	20.83 (11.01–30.64)	*p* < 0.001	*I* ^2^ = 10.6%, *p* = 0.348	0.289	0.188
	N 1.0 mm/6	36.95 (20.52–53.38)	*p* < 0.001	*I* ^2^ = 57.3%, *p* = 0.039	0.020	0.851
	S 1.0 mm/2	31.66 (12.50–50.83)	*p* = 0.001	*I* ^2^ = 0.0%, *p* = 0.821	—	0.317
	I 1.0 mm/2	31.34 (13.92–48.76)	*p* < 0.001	*I* ^2^ = 0.0%, *p* = 0.775	—	0.317
	T 2.0 mm/2	7.71 (−43.79–59.21)	*p* = 0.769	*I* ^2^ = 75.5%, *p* = 0.044	—	0.317
	N 2.0 mm/2	45.63 (21.18–70.09)	*p* < 0.001	*I* ^2^ = 0.0%, *p* = 0.908	—	0.317
	T 3.0 mm/2	17.46 (2.59–32.33)	*p* < 0.001	*I* ^2^ = 24.3%, *p* = 0.250	—	0.317
	N 3.0 mm/2	26.81 (13.68–39.93)	*p* = 0.021	*I* ^2^ = 0.0%, *p* = 0.593	—	0.317

A versus C	T 0.5 mm/2	74.12 (16.74–131.50)	*p* = 0.011	*I* ^2^ = 79.9%, *p* = 0.026	—	0.317
	N 0.5 mm/2	30.71 (−7.14–74.56)	*p* = 0.106	*I* ^2^ = 58.4%, *p* = 0.121	—	0.317
	T 1.0 mm/6	29.01 (15.01–49.00)	*p* < 0.001	*I* ^2^ = 44.3%, *p* = 0.110	0.175	0.851
	N 1.0 mm/6	55.05 (28.60–81.50)	*p* < 0.001	*I* ^2^ = 84.4%, *p* < 0.001	0.478	0.573
	S 1.0 mm/2	50.92 (−6.18–108.03)	*p* = 0.080	*I* ^2^ = 82.4%, *p* = 0.017	—	0.317
	I 1.0 mm/2	47.42 (3.89–90.94)	*p* = 0.033	*I* ^2^ = 65.8%, *p* = 0.087	—	0.317
	T 2.0 mm/2	24.26 (−1.59–50.11)	*p* = 0.066	*I* ^2^ = 0.0%, *p* = 0.392	—	0.317
	N 2.0 mm/2	67.60 (3.77–91.43)	*p* < 0.001	*I* ^2^ = 0.0%, *p* = 0.336	—	0.317
	T 3.0 mm/2	20.12 (10.06–30.17)	*p* < 0.001	*I* ^2^ = 0.0%, *p* = 0.882	—	0.317
	N 3.0 mm/2	27.01 (13.65–40.38)	*p* < 0.001	*I* ^2^ = 0.0%, *p* = 0.548	—	0.317

F Vs C	T 0.5 mm/2	35.06 (−36.61–106.73)	*p* = 0.338	*I* ^2^ = 89.1%, *p* = 0.002	—	0.317
	N 0.5 mm/2	−9.35 (−34.53–15.84)	*p* = 0.467	*I* ^2^ = 16.4%, *p* = 0.274	—	0.317
	T 1.0 mm/6	5.86 (−12.10–23.81)	*p* = 0.523	*I* ^2^ = 68.7%, *p* = 0.007	0.917	0.851
	N 1.0 mm/6	17.13 (−1.09–35.34)	*p* = 0.065	*I* ^2^ = 69.0%, *p* = 0.006	0.761	0.573
	S 1.0 mm/2	19.54 (−33.19–72.28)	*p* = 0.468	*I* ^2^ = 79.1%, *p* = 0.029	—	0.317
	I 1.0 mm/2	15.64 (−32.93–64.21)	*p* = 0.528	*I* ^2^ = 73.6%, *p* = 0.052	—	0.317
	T 2.0 mm/2	16.26 (−13.02–45.54)	*p* = 0.276	*I* ^2^ = 24.2%, *p* = 0.251	—	0.317
	N 2.0 mm/2	18.37 (−6.87–43.61)	*p* = 0.154	*I* ^2^ = 18.7%, *p* = 0.267	—	0.317
	T 3.0 mm/2	4.75 (−3.41–12.91)	*p* = 0.254	*I* ^2^ = 4.3%, *p* = 0.307	—	0.317
	N 3.0 mm/2	0.98 (−12.55–14.51)	*p* = 0.003	*I* ^2^ = 0.0%, *p* < 0.001	—	0.317

T: temporal; N: nasal; S: superior; I: inferior; WMD: weighted mean differences; CI: confidence interval; A: amblyopia; C: control; F: fellow.

## Abbreviations

AHRQ: Agency for Healthcare Research and Quality
AL: Axial length
CI: Confidence interval
CT: Choroidal thickness
EDI-OCT: Enhanced depth imaging optical coherence tomography
OCT: Optical coherence tomography
SFCT: Subfoveal choroidal thickness.

## Additional Points

*Availability of Data and Materials*. All data generated or analyzed during this study are included in this published article and its additional files.
