# Supplementary material for: A Meta-Analysis of Choroidal Thickness Changes in Unilateral Amblyopia
Source: J Ophthalmol. 2017 Jun 19;2017:2915261. doi: 10.1155/2017/2915261 (PMC5494565; doi:10.1155/2017/2915261)
Supplement: Supplementary file 2 [file 2915261.f2.docx]

**S2 Fig. search strategy (PubMed)**

1. “choroidal”[All Field]

2. “choroid”[All Field]

3. 1 or 2

4. “optical coherence tomography”[All Field]

5. “spectral-domain optical coherence tomography” [All Field]

6. “spectral domain optical coherence tomography” [All Field]

7. 4 or 5 or 6

8. “Animals” [All Field]

9. “Human”[All Field]

10. 9 not 8

11. “Thickness”[All Field]

12. “Amblyopia”[All Field]

13. 3 and 7and 10 and 11 and 12
